# Molecular Landscape of Modified Histones in *Drosophila* Heterochromatic Genes and Euchromatin-Heterochromatin Transition Zones

**DOI:** 10.1371/journal.pgen.0040016

**Published:** 2008-01-18

**Authors:** Jiro C Yasuhara, Barbara T Wakimoto

**Affiliations:** Department of Biology, University of Washington, Seattle, Washington, United States of America; University of Cambridge, United Kingdom

## Abstract

Constitutive heterochromatin is enriched in repetitive sequences and histone H3-methylated-at-lysine 9. Both components contribute to heterochromatin's ability to silence euchromatic genes. However, heterochromatin also harbors hundreds of expressed genes in organisms such as *Drosophila*. Recent studies have provided a detailed picture of sequence organization of D. melanogaster heterochromatin, but how histone modifications are associated with heterochromatic sequences at high resolution has not been described. Here, distributions of modified histones in the vicinity of heterochromatic genes of normal embryos and embryos homozygous for a chromosome rearrangement were characterized using chromatin immunoprecipitation and genome tiling arrays. We found that H3-di-methylated-at-lysine 9 (H3K9me2) was depleted at the 5′ ends but enriched throughout transcribed regions of heterochromatic genes. The profile was distinct from that of euchromatic genes and suggests that heterochromatic genes are integrated into, rather than insulated from, the H3K9me2-enriched domain. Moreover, the profile was only subtly affected by a *Su(var)3–9* null mutation, implicating a histone methyltransferase other than SU(VAR)3–9 as responsible for most H3K9me2 associated with heterochromatic genes in embryos. On a chromosomal scale, we observed a sharp transition to the H3K9me2 domain, which coincided with increased retrotransposon density in the euchromatin-heterochromatin (eu-het) transition zones on the long chromosome arms. Thus, a certain density of retrotransposons, rather than specific boundary elements, may demarcate *Drosophila* pericentric heterochromatin. We also demonstrate that a chromosome rearrangement that created a new eu-het junction altered H3K9me2 distribution and induced new euchromatic sites of enrichment as far as several megabases away from the breakpoint. Taken together, the findings argue against simple classification of H3K9me as the definitive signature of silenced genes, and clarify roles of histone modifications and repetitive DNAs in heterochromatin. The results are also relevant for understanding the effects of chromosome aberrations and the megabase scale over which epigenetic position effects can operate in multicellular organisms.

## Author Summary

The chromosomal domain “heterochromatin” was first defined at the cytological level by its deeply staining appearance compared to more lightly stained domains called “euchromatin.” Abnormal juxtaposition of these two domains by chromosome rearrangements results in silencing of the nearby euchromatic genes. This effect is mediated by heterochromatin-enriched chromosomal proteins and led to the prevalent view of heterochromatin as incompatible with gene expression. Paradoxically, some expressed genes reside within heterochromatin. In this study, we examined how heterochromatic genes fit into a genomic context known for silencing effects. We found that *Drosophila* heterochromatic genes are integrated into the domain enriched in the modified histone H3K9me2, suggesting that the effect of this protein on gene expression is context-dependent. We also investigated the molecular nature of euchromatin-heterochromatin transition zones in the normal and rearranged chromosomes. The results provide insights into the functions of repetitive DNAs and H3K9me2 in heterochromatin and document the long distance over which a heterochromatic breakpoint can affect the molecular landscape of a chromosomal region. These findings have implications for understanding the consequences of chromosome abnormalities in organisms, including humans.

## Introduction

Constitutive heterochromatin is a nearly universal component of eukaryotic genomes. It was first defined in the 1920′s as distinct from euchromatin by its densely stained cytological appearance [1]. It was also associated with modulation of gene expression in *Drosophila* chromosome rearrangements that created new euchromatin-heterochromatin (eu-het) junctions [2,3]. The variable silencing of euchromatic genes located near the eu-het breakpoint was described as position effect variegation (PEV). Striking aspects of PEV included its generality and long-range effects on genes located as far as several megabases away from the breakpoint. Decades later, it was recognized that constitutive heterochromatin is mostly composed of repetitive sequences, including transposable elements (TEs) [4] and satellite sequences [5], and enriched for certain chromosomal proteins, particularly the chromodomain protein HP1 and specific modified forms of histones, such as H3K9me2 or H3K9me3 (histone H3-di, or tri-methylated-at-lysine 9) [6]. Mutations that disrupt the function of these and other heterochromatin-enriched proteins or histone modifying enzymes have been identified as Suppressors of PEV (*Su(var)s*) [7] in *Drosophila* because of their effects on heterochromatin-induced PEV of euchromatic genes. Many SU(VAR) proteins are evolutionarily conserved among eukaryotes, including yeast and humans.

The fission yeast, *Schizosaccharomyces pombe,* lacks cytologically defined heterochromatin but its pericentromeric and silent mating-type regions share genetic and biochemical properties, including an enrichment for H3K9me, with the heterochromatin of multicellular eukaryotes. Elegant studies using S. pombe reported two key observations regarding the nucleation and delimitation of H3K9me-enriched domains. First, distinct repetitive DNA elements produce double-stranded RNAs, which are processed into small RNAs and mediate recruitment and assembly of complexes containing the RNAi machinery, H3K9 methyl-transferase, and HP1 homologs [6,8–11]. Second, distinct boundary elements exist that limit spreading of the complexes from nucleation sites [11,12]. In the silent mating-type region, for example, there is a dramatic transition between H3K4me (histone H3 methylated-at-lysine 4)-domains that contain active genes and H3K9me-domains that contain silent loci, precisely at boundary elements. Deletion of these boundary elements results in leakage of H3K9me from silent domains into neighboring domains over distances of several kilobases [12].

The discovery of small RNAs derived from TE DNAs [13] and effects of mutations in the RNAi machinery on PEV [14] has led some investigators to propose a role for RNAi in the formation of *Drosophila* heterochromatin [14]. Whether other properties observed for S. pombe may extend to the much larger and more complex heterochromatin of multicellular organisms remains unclear. Heterochromatin can encompass many megabases of DNA in plants and animals, and its influence can extend several megabases into euchromatin [2,3,7,15]. Moreover, heterochromatin contains a complex repertoire and distribution of repetitive elements in organisms such as *Drosophila*. Little is known about the relative contributions of various types of repetitive elements to heterochromatin formation, although a few studies in *Drosophila* have singled out the *1360* element as a prime candidate for Chromosome 4 heterochromatin [16]. In addition, the molecular nature of eu-het transition zones remains largely unexplored.

The association of heterochromatin, heterochromatin-enriched proteins, and gene silencing is widely accepted [7], yet hundreds of genes are embedded within heterochromatin in diverse organisms [17,18]. At least some of these genes in *Drosophila* depend on a heterochromatic location and associated chromosomal proteins for normal expression [18]. Compelling evidence is provided by studies of chromosome rearrangements that create new eu-het junctures and result in repressive effects on both euchromatic genes and heterochromatic genes on either side of the breakpoint [3,19–21]. In addition, genetic studies have shown that a large number of modifiers of PEV (*Su(var)*s and *E(var)*s) act in opposite fashions on variegating euchromatic and heterochromatic genes [22].

Several models have been proposed to account for the expression of heterochromatic genes [18]. The simplest is an “insulation” model which proposes that genes located within cytologically defined heterochromatin actually exist in isolated “islands” of euchromatin and are protected from the surrounding repressive chromatin. This model cannot account for genes that depend on heterochromatin for expression. An “exploitation” model does so, by postulating that heterochromatic genes possess specific sequences, for example specialized promoters or TE-derived regulatory sequences, which require factors enriched in or limited to heterochromatin. In previous study, we eliminated the possibility of novel types of promoter for seven of the best studied *Drosophila* heterochromatic genes [23]. A third model, “integration,” proposes that heterochromatin-enriched proteins act more broadly to set up an environment that promotes heterochromatic gene expression. One mode of action may be to facilitate long-range enhancer-promoter interactions through protein-protein interactions. Implicit in this model is the view that heterochromatin-enriched proteins and sequences are multifunctional, with positive, negative, or neutral activities being context dependent. Thus heterochromatic genes may be integrated into a domain, which while incompatible for euchromatic genes, is nonetheless required for efficient expression of heterochromatic genes.

To evaluate models of heterochromatic gene expression, we investigated the distribution of H3K9me and other modified histones in and around heterochromatic genes in *Drosophila* embryos. We also investigated whether SU(VAR)3–9, which has been characterized as the histone methyltransferase (HMTase) primarily responsible for H3K9me in pericentric heterochromatin [24], affects chromatin modifications of heterochromatic genes. Our analysis provides the first large-scale view of the molecular landscape of modified histones in *Drosophila* heterochromatic genes and naturally occurring and rearrangement-induced euchromatin-heterochromatin transition zones. The findings are relevant for considering how modified histones and repetitive DNAs contribute to the specification and influence of heterochromatic domains.

## Results

### Distribution of Modified Histones in Heterochromatic Genes

Specific lysines in the tail of histone H3, including Lys 4, 9, 27, and 36, can be mono-, di-, or tri-methylated. Of the many known H3 methylation states, methylation of Lys 9 (H3K9me) has been most extensively studied and has been strongly correlated with gene repression and establishment of heterochromatin [25]. Previous studies using specific antibodies to detect modified H3 on polytene chromosomes showed that H3K9-di-methylation (H3K9me2) is the major H3K9 modification in *Drosophila* pericentric heterochromatin [24,26,27]. Thus, we focused on H3K9me2. We also assayed H3 di-methylated at Lys 4 (H3K4me2) and H3 acetylated at Lys 9 and 14 (H3K9/14acet) because they are believed to mark gene expression in euchromatin [28–30] but their presence and distribution in *Drosophila* heterochromatin have not been reported.

We first determined modified histone distributions in three protein-encoding genes, *light* (*lt*), *concertina* (*cta*), and *Chitinase*, which are located in Chromosome 2L heterochromatin (2Lh). We assayed chromatin of postblastoderm embryos (4–14 h) using chromatin immunoprecipitation (ChIP), a technique that reports the average chromatin profile for the population of nuclei assayed. Although these embryos contained multiple tissue types, previous studies showed that *lt* and *cta* are widely expressed throughout development, including during embryogenesis [31–33], and ubiquitous expression has been documented in larval polytene and diploid nuclei by RNA *in situ* hybridization [34]. Because maternal loading of transcript obscured detection of zygotic transcription in earlier studies [32,33], we verified transcription of *lt* and *cta* in 4–14 h embryos using a nuclear run-on assay (Figure S1). This assay also verified little or no expression of the *Chitinase* gene in 4–14 h embryos, a result consistent with previous conclusions that this gene functions mainly in larval molting [35].

**Figure 1:**
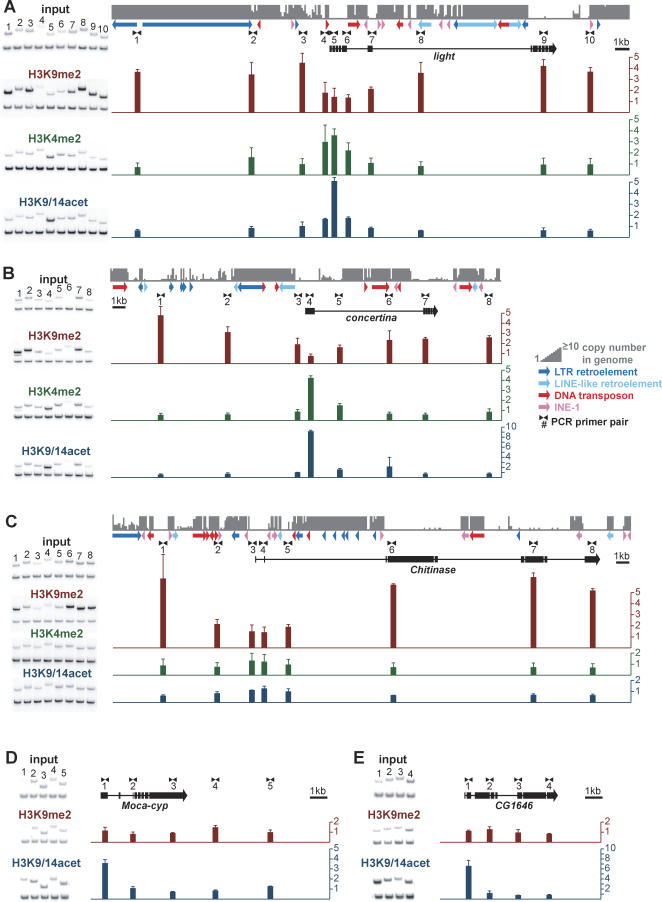
Distribution of Modified Histones in Representative Heterochromatic and Euchromatic Genes (A) ChIP results for the *light* gene. The top diagram summarizes gene structure, using the key shown in the figure. Gray blocks on the top line indicate whether the region is repetitive (high bar) or unique (no bar). For this diagram, non-overlapping 100 bp windows were individually BLASTed against the whole genome sequence and each hit with E < 10^−3^ was counted as one copy. The colored arrows show orientation and types of transposable elements located in the repetitive regions (INE-1 is a highly abundant TE that constitutes its own category). The black arrow depicts the exons (black blocks) and introns (black lines). Pairs of opposing black triangles with identification numbers show the locations of ten PCR primer pairs used in the ChIP assay. Left panels show the gel assay of quantitative duplex PCR of input or ChIP samples as indicated, with the numbers identifying the corresponding PCR primer pair used for each lane. The common bottom band in each lane is the normalization control *Pdi* (Materials and Methods). The bar graphs summarize ChIP results across the length of the gene (*x*-axis), with bar heights showing average enrichment factors (*y*-axis) and standard errors for three trials for H3K9me2 (top graph), and two trials each for H3K4me2 (middle) and H3K9/14acet (bottom). ChIP results for (B) *concertina* and (C) *Chitinase* with the same number of trials as described for *lt*. ChIP results for two euchromatic control genes, (D) *Moca-cyp* and (E) *CG1646* with two trials each.

The exons of the 2Lh genes are unique sequence, but the bulk of introns and flanking sequences consist of repetitive TE-like sequences (Figure 1). This structure required careful design of PCR primer pairs for the ChIP assay since detection of recovered fragments is hybridization-based. PCR primer pairs were designed so: (i) at least one primer in the pair was unique sequence, or (ii) both primers in the pair were different repetitive sequences but the juxtaposition of the two sequences was a unique occurrence in the genome. There were three instances in the case of the *lt* gene in which both primers in a pair were anchored in repetitive sequences. We verified specificity of these primer sets by showing that PCR fragments were amplified from DNA of normal embryos, but not from embryos deleted for the *lt* gene region (Figure S2). This scheme allowed specific detection of 2Lh gene sequences, despite the fact that most regions are repeated many times in the genome.

Chromatin was immunoprecipitated with antibodies specific for H3K9me2, H3K4me2, or H3K9/14acet, then assayed for heterochromatic gene sequences and a control euchromatic gene sequence (*Pdi)* used for normalization. Enrichment was expressed relative to input DNA. These ChIP assays showed that H3K9me2 was enriched in the *lt* and *cta* genes and distributed across most of the transcribed regions except for the 5′-ends (Figure 1A and 1B). Depletion of H3K9me2 in the transcription start-regions was complemented by enrichment of H3K4me2 and H3K9/14acet. A peak of H3K4me2 and H3K9/14acet in the start-region seems to be a universal feature of expressed genes in higher eukaryotes [28–30], and *in vitro* studies indicate that these modifications and H3K9me may be inhibitory to each other in individual histone molecules [36,37]. However, depletion of H3K9me2 in the start-region may not necessarily be due to exclusion by the antagonistic histone modifications. This is indicated by the modified histone distribution in the *Chitinase* gene, which lacks H3K9me2 as well as H3K4me2 and H3K9/14acet at the start-region (Figure 1C). This result is consistent with minimal *Chitinase* transcription in embryos (Figure S1).

We also examined three euchromatic genes, *Moca-cyp*, *CG1646,* and *CG551*4, which previous *in situ* RNA hybridization and microarray studies showed are highly and ubiquitously expressed in 4–14 h embryos [38]. All three genes were enriched for H3K9/14acet at the 5′ end, but lacked significant levels of H3K9me2 (Figure 1D and 1E; *CG5514* data not shown). The general lack of H3K9me2 in euchromatic genes was also evident in a larger scale ChIP-chip analysis described below. Thus, heterochromatic and euchromatic genes carried similar H3K9/14acet signatures of expression, but differed in H3K9me2 association. Distribution of H3K9me2 throughout heterochromatic genes indicated integration into, rather than insulation from, the surrounding H3K9me2-enriched environment.

### Global Landscape of Modified Histones in Heterochromatic Genes

**Figure 2:**
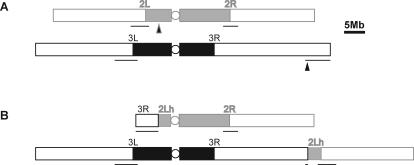
Schematic View of D. melanogaster Major Autosomes, Chromosomes 2 and 3 (A) The chromosomes of a wild-type strain showing location of heterochromatin (filled rectangles), euchromatin (open rectangles) on left (L) and right (R) arms, centromeres (circle), and regions (underlined) that were included in the tiling microarray. The triangles indicate the positions of the breakpoints in the *T(2;3)lt^x13^* translocation. (B) Structure of the *T(2;3)lt^x13^* translocation. Heterochromatin of 2L is denoted 2Lh.

To obtain global perspectives of H3K9me2 and H3K9/14acet profiles in heterochromatin, we used a ChIP-chip approach. The genome tiling array contained probes for heterochromatic regions of the genome, as assembled and annotated in the D. melanogaster genome project Release 5.1 (R5.1) [39,40]. Specifically, we focused on the proximal ends of the assembled genome sequence corresponding to the euchromatin-heterochromatin (eu-het) transition zones and extending into distal heterochromatin of the long 2L, 2R and 3L chromosome arms (Figure 2A). We also included a segment of 3R distal euchromatin and the entire sequenced region of the small Chromosome 4 for comparisons among chromosomal regions. To circumvent the cross-hybridization problem of repetitive sequences, genome sequence was masked for annotated TEs and remaining sequences were used to design overlapping 50-mer oligonucleotides probes with 40 bp resolution. This probe set was subject to stringent filtering to eliminate repetitive sequences, and remaining probes were used for the microarray (Materials and Methods). Probe density necessarily varied across heterochromatin due to repeat filtering.

**Figure 3:**
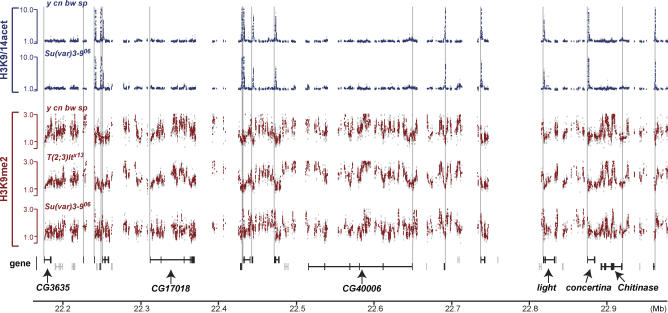
Modified Histone Profiles of the 2Lh Genes Revealed by ChIP-chip Assays Comparison of H3K9/14acet (top) and H3K9me2 (below) profiles for the genotypes indicated at the top of each line shows the reproducibility of the profiles. ChIP-enrichment relative to total genomic DNA control is plotted. The raw data are shown as gray dots, and averages of five adjacent probes are shown as colored dots (blue for H3K9/14acet and brown for H3K9me2). The annotated heterochromatic genes are shown below with the depiction of exon/intron structures. Genes on the plus and minus strands are shown in the top and the bottom tiers, respectively. Black genes have cDNA evidence to support their existence and the positions of their annotated 5′ ends are marked by the gray vertical lines. Gray genes are based solely on computational predictions. *x*-Axis shows the R5.1 genome coordinates.

**Figure 4:**
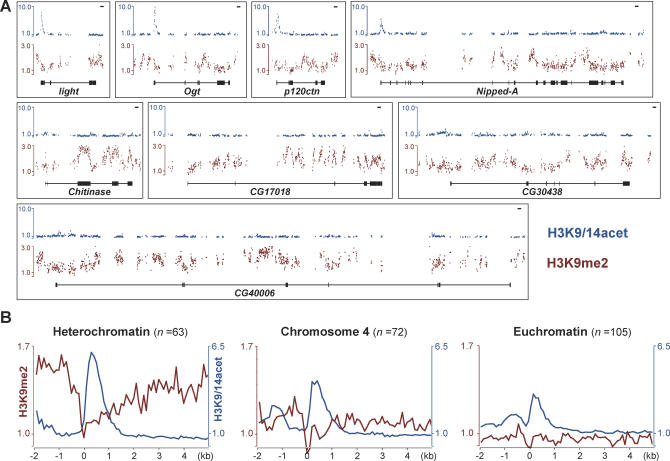
Modified-Histone Profiles of Heterochromatic Genes as Revealed by ChIP-chip Assays (A) Each panel shows a single gene with profiles of H3K9/14acet-enrichment (top, blue), H3K9me2-enrichment (middle, brown), and gene structure depicted in 5′ → 3′ orientation (bottom, black). Scale bar (line in upper right) is 1 kb. (B) Each graph shows the average H3K9/14acet (blue, right *y*-axis) and H3K9me2 (brown, left *y*-axis) profiles of genes located in either in heterochromatin, Chromosome 4, or euchromatin of 3R, with *n* indicating number of genes in each class. To facilitate the analysis of euchromatic genes, only genes on the plus strand in a 3 Mb region of Chromosome 3R (coordinate 22.5–25.5Mb) were included. Gene sequences were aligned at the annotated 5′-ends (0 on the *x-*axis), broken into 100 bp windows, and the enrichment factors for all probes included in each window were averaged and plotted.

Reproducible H3K9/14acet and H3K9me2 profiles were obtained in the ChIP-chip studies (Figure 3), permitting a comparison with the standard ChIP results (Figure 1). Specifically, the ChIP-chip assay showed strong H3K9/14acet enrichment in the 5′ regions of heterochromatic genes, but did not indicate as high an enrichment of H3K9me2 as anticipated based on standard ChIP. Nonetheless, the observed pattern was consistent with earlier results. As shown for 2Lh in Figure 3, H3K9me2 was enriched in intergenic regions and throughout these heterochromatic genes but not near the transcription start-regions. This was also true for genes with little or no association with H3K9/14acet (e.g., *Chitinase*, *CG17018,* and *CG40006* genes in Figures 3 and 4A).

To obtain an average profile of the heterochromatic genes represented on our tiling array, we defined a set of genes as heterochromatic if they were located proximal to the *CG3635*, *nrm*, or *CG11665* genes at the base of 2L, 3L, or 2R respectively. Of the 111 annotated genes that met this criterion, 63 had cDNA evidence to support the annotation of the 5′ end. As shown in Figure 4B, the average modified histone profiles of these 63 genes differed from those for two other categories of genes included in our microarray: protein-encoding genes located on Chromosome 4 (*n* = 72) and euchromatic genes located on distal 3R (*n* = 105). All three classes showed a peak of H3K9/K14acet at the 5′ start region with differences in peak size likely reflecting the different proportion of genes expressed in embryos in each gene set. Based on recovery of embryo ESTs reported in FlyBase [41], 81% of the genes in the heterochromatic gene set, 71% of the Chromosome 4 genes, and 55% of the 3R-euchromatic genes are transcribed in embryos. Notably, heterochromatic genes showed a significantly higher average level of H3K9me2 compared to Chromosome 4 and 3R euchromatic genes (Figure 4B). Overall, Chromosome 4 genic and intergenic regions exhibited slightly elevated average levels of H3K9me2 relative to those typically observed within euchromatin (Figure S3).

### A *Su(var)3–9* Null Mutation Has a Subtle Effect on H3K9me2 Profiles of Heterochromatic Genes

**Figure 5:**
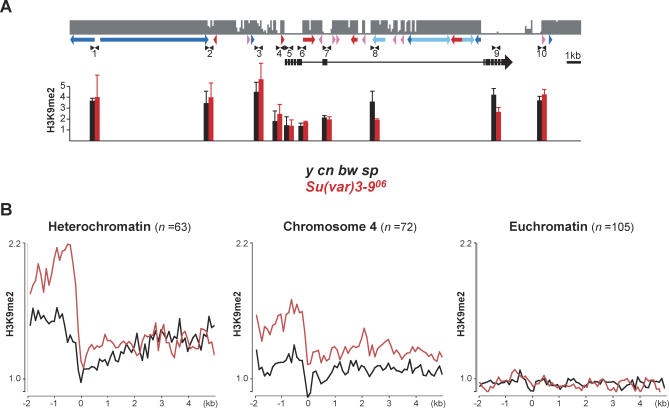
Comparison of H3K9me2 Profiles in Wild-type and *Su(var)3–9^06^* Embryos (A) The bar graph compares the H3K9me2 enrichment factors in the *light* gene of *y; cn bw sp* (black) and *Su(var)3–9^06^* (red) embryos based on three and two trials, respectively. Symbols are the same as in Figure 1. (B) Comparison of the average H3K9me2 profile of heterochromatic genes (*n* = 63), Chromosome 4 genes (*n* = 72), and euchromatic genes (*n* = 105) in the two genotypes, plotted as described for Figure 4B.

To determine if the SU(VAR)3–9 HMTase was responsible for the H3K9me2 enrichment in and around heterochromatic genes, we examined the effects of a null mutation *Su(var)3–9^06^* [24]. The ChIP assay revealed small but reproducible differences between the wild type and homozygous *Su(var)3–9^06^* in the relative levels of H3K9me2 across the *lt* gene (Figure 5A). To further investigate this effect, we performed two separate ChIP-chip hybridization comparisons of wild type and *Su(var)3–9^06^* embryos. We found that the landscape of H3K9/K14acet and H3K9me2 across 2Lh (Figure 3) and other heterochromatic regions (data not shown) appeared overall similar in the two genotypes. However, in the mutant embryos, the average profile of heterochromatic genes showed a slightly elevated level of H3K9me2 just upstream of the start site relative to the level observed within the transcribed region (Figure 5B). The average profile across euchromatic genes was not detectably altered in the mutant. A striking difference between the two genotypes was evident from examining H3K9me2 distribution on Chromosome 4. Relative to wild type embryos, *Su(var)3–9^06^* embryos showed a higher average level of H3K9me2 within Chromosome 4 genes and more pronounced regional increases along the length of the chromosome (Figure S3). This unexpected observation of localized increases, rather than reductions of H3K9me2 associated with *Su(var)3–9^06^* indicates that in embryos, SU(VAR)3–9 is not responsible for the majority of H3K9me2 in the regions we assayed here, but it apparently influences one or more HMTases that play more prominent roles.

### Global Landscape of H3K9me2 in the Euchromatin-Heterochromatin Transition Zones

**Figure 6:**
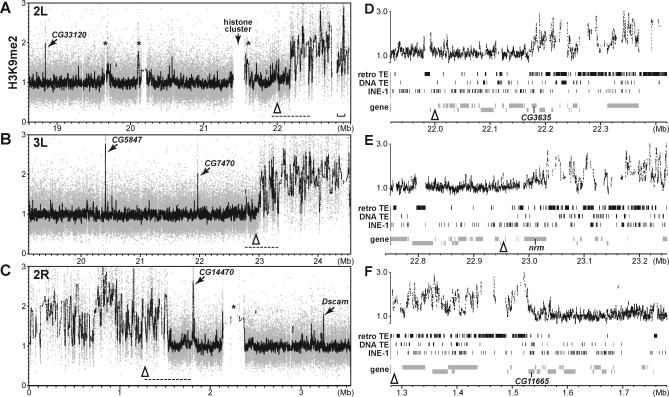
H3K9me2 Landscapes in the Euchromatin-Heterochromatin Transition Zones (A–C) H3K9me2-enrichment relative to the genomic DNA reference control in the chromosome arms 2L (A), 3L (B), and 2R (C). Each panel corresponds to approximately one-sixth of the assembled genome sequence located at the proximal base of the chromosome arm (Figure 2A). Raw data are shown in gray and moving average values of 50 adjacent probes are shown in black. The R5.1 genomic coordinates are shown on the *x*-axes. Open triangles note locations of the R5.1 heterochromatin sequence reference points which are reported in FlyBase and were designated as heterochromatic by Smith *et al.* [39] based on chromosomal fluorescent *in situ* hybridization localizations. The asterisks indicate euchromatic regions with high TE-density. The bracket in (A) marks the interval containing *lt*, *cta*, and *Chitinase* genes. (D–F) Expanded views of the transition zones whose locations are marked by the dashed lines in (A–C). In each panel, the H3K9me2-enrichment values (moving average of ten adjacent probes) are shown in upper diagram and distributions of TEs, and genes are shown below. Genes are depicted in two tiers to distinguish 5′ to 3′ orientations. Apparent overlap among TEs and between TEs and genes are due to nesting events. Locations of the *CG3635*, *nrm*, and *CG11665* genes, which are located at the abrupt transitions to the enriched H3K9me2 domains at the base of 2L, 3L, or 2R respectively, are noted.

The H3K9me2 profile characteristic of the long autosomal arms differed markedly from that observed for Chromosome 4. In euchromatic regions, the profile was typically flat (Figures S3 and 6). However, within the eu-het transition zones on 2L, 2R, and 3L, a sharp transition was observed between low levels of H3K9me2 in distal regions to enriched domains more proximally (Figure 6). Sequence inspection did not reveal common underlying sequences at the transition point, or conspicuous features such as large inverted repeats or tRNA genes that characterize the boundary elements of yeast silent chromatin domains [12,42]. However, the transitions coincided with increased density of retrotransposons including LTR-type and LINE-like TEs (Figure 6D–6F).

Within the limited coverage of ∼15 Mb of euchromatin on the tiling array, there was no significant association of H3K9me2 noted in sequences adjacent to the many solitary TEs in euchromatin. However, we observed several prominent peaks that corresponded to the coding sequences of unique genes (Figures 6A–6C and S4). A few less prominent sites resided adjacent to regions with a high TE density (Figure 6A–6C, asterisks) and while these were reproducible, they showed overall lower and more local H3K9me2 enrichment compared to the broad peaks evident in heterochromatin.

### H3K9me2 Profiles of TEs

Included on the tiling array were probes for sequences contained in 106 “canonical” TEs. These sequences represented full-length consensus sequences of each TE type [43] and were included to ask whether certain classes of TEs might exhibit overall higher associations with H3K9me2. The interpretation of the ChIP-chip data for these sequences may be complicated by differences in TE copy numbers in the genome and the proportion of complete vs. incomplete versions which vary with different elements. Nonetheless, each TE generated a distinctive H3K9me2 profile that was reproducible in two different genotypes (Figure S5; Table S1). When TEs were ranked by the average H3K9me2-enrichment across their lengths, it was clear that the higher-ranked TEs belonged to retrotransposon class. Forty-eight TEs that showed the highest average H3K9me2-enrichment were all retrotransposons. DNA transposons, such as the *1360* element, show moderate to low H3K9me2-enrichment in this assay. Another notable observation was that TEs with especially high copy number in euchromatic regions [43], such as the *roo* element, tended to show lower H3K9me2-enrichment.

### Rearrangement Induced Changes in H3K9me2 Distribution

We designed the genomic tiling array to investigate whether a chromosome rearrangement which created a new eu-het junction might be associated with detectable changes in H3K9me2 distribution. We compared embryos homozygous for *T(2;3)lt^x13^*, a reciprocal translocation which displaces most of 2Lh distally to 3R [19] (Figure 2B), to two wild-type strains: *Canton-S*, which was the parent strain for *T(2;3)lt^x13^* and *y; cn bw sp*, the strain used for the genome sequencing and annotation projects.

**Figure 7:**
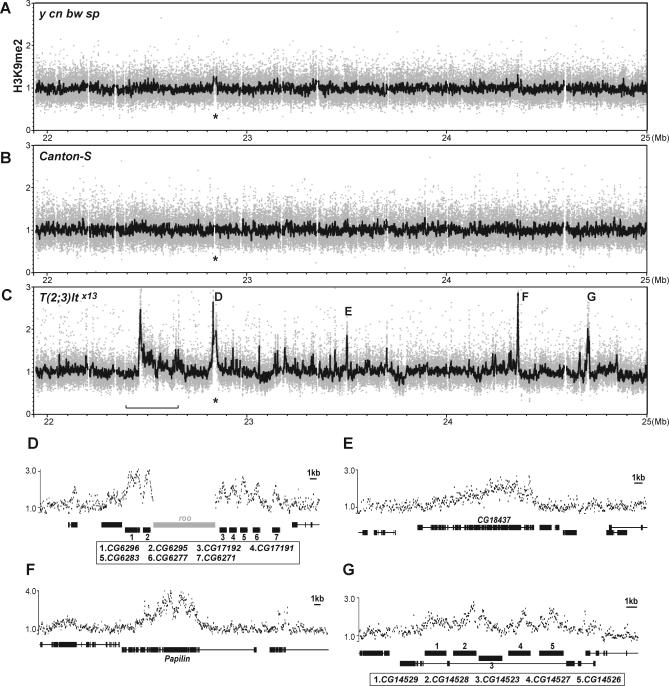
Alteration of H3K9me2 Profile by Chromosome Rearrangement (A–C) The plots show the H3K9me2-profiles for Chromosome 3R euchromatic region, which is underlined in Figure 2. This region is located distally in 3R in normal strains *y; cn bw sp* (A) and *Canton-S* (B) and displaced proximally in the rearranged chromosome *T(2;3)lt^x13^* (C) . The bracket in (C) indicates the 97C region where the *T(2;3)lt^x13^* breakpoint has been localized [19]. The gap in the probes marked by asterisk corresponds to the *roo* retrotransposon mentioned in the text. (D–G) Expanded views of *T(2;3)lt^x13^*-induced H3K9me2 peaks. Peaks at coordinates 23.5 Mb (E) and 24.3–24.4 Mb (F) coincide with a large gene with high exon density. Peaks at 22.8–22.9 Mb (D) and 24.7 Mb (G) coincide with a duplicated gene cluster. In (D), these genes are 58%–93% identical to each other at the nucleotide sequence level over an ∼1 kb stretch. In (G), *CG14527* and *CG14526* are 84% identical over ∼2kb. However, the other gene pairs in this cluster are highly diverged (<55% identity at the nucleotide sequence level, ∼50% similarity at the amino-acid sequence level).

Both halves of the translocation are of interest to assay for H3K9me2 profiles. Flies heterozygous for *T(2;3)lt^x13^* and chromosomes that carry null alleles of the 2Lh genes have pronounced variegated phenotypes for *lt* and other 2Lh genes. However, flies homozygous for *T(2;3)lt^x13^* are viable and show very weak variegation of the *lt* gene in adult tissues [19]. As shown in Figure 3, the ChIP-chip assays did not reveal differences in the H3K9me2 profile of the distally displaced 2Lh genes in embryos homozygous for *T(2;3)lt^x13^* compared to wild-type embryos. However, significant changes were observed in the 3R euchromatic segment that was placed adjacent to pericentric heterochromatin (Figure 7). Euchromatic sequences adjacent to the breakpoint showed an abrupt enrichment of H3K9me2, with continued overall enrichment extending at least 200 kb (from coordinate 22.4–22.6 Mb), reflecting spreading of the H3K9me2 domain across the breakpoint. Also striking were many discrete H3K9me2 peaks scattered over a ∼3 Mb region. The most prominent peaks were found over coding sequences of genes (Figure 7D–7G). Some, but not all, of the peaks may have resulted from enhancement of H3K9me2 affinity sites present on normal chromosomes. For example, a *roo* element is present at coordinate 22.8–22.9 Mb in the *y; cn bw sp* strain and adjacent single copy sequences on either side have a slightly higher than average H3K9me2 association (Figure 7A). It is possible that the prominent peak in *T(2;3)lt^x13^* is formed around a *roo* element in this same location (Figure 7C and 7D). Perhaps more relevant is that this *roo* element is nested in a cluster of seven duplicated genes (Figure 7D). Another example of possible enhancement of a site showing H3K9me2 affinity is located at coordinates 24.3–24.4 Mb which corresponds to the coding sequence of the *Papilin* gene (Figure 7F). Other peaks in *T(2;3)lt^x13^* had no apparent presages in the normal chromosomes but notably, the site at coordinate 24.7 Mb encompassed cluster of five duplicated genes (Figure 7G). Additional peaks near the telomere were also observed in *T(2;3)lt^x13^* but not normal chromosome (data not shown). These results demonstrate that a chromosome rearrangement can exert an epigenetic effect as far as 5 Mb distance from the breakpoint. In addition, the H3K9me2 profile in *T(2;3)lt^x13^* was noticeably “bumpy” in the displaced 3R compared to the flat profile in the corresponding regions in normal chromosomes (Figure 7), suggesting a pervasive H3K9me2 association on this short chromosome arm.

## Discussion

*Drosophila* has traditionally provided a powerful, genetically tractable system for studies of heterochromatin. Here we combined genetic tools, improved heterochromatin sequence coverage, and ChIP approaches to obtain a high-resolution and large-scale molecular view of modified histone distribution in *Drosophila* heterochromatin. We used embryos, rather than cultured cells, for chromatin profiling to examine the effects of mutations or chromosome rearrangements whose consequences in the whole organism have been well documented.

Analysis of the distribution of modified histones showed that 5′ regions of expressed heterochromatic genes were enriched for H3K4me2 and H3K9/14acet, consistent with these modifications serving as markers of active genes in both heterochromatin and euchromatin [28–30]. The complementary distributions of H3K4me2, H3K9/14acet, and H3K9me2 support previous findings that these are mutually exclusive H3 modifications [36,37]. Most significantly, we found that H3K9me2 enrichment throughout transcribed regions is a general property of heterochromatic genes. Hence, heterochromatic genes constitute a category of genes that are an integral part of the H3K9me-enriched domain and are not insulated from it. This contrasts with the situation for *Arabidopsis* genes that are located in a heterochromatic “knob”, since the knob region is highly enriched for H3K9me and DNA-methylation as a whole, but expressed genes are largely free of these molecular marks [44].

Our results showing H3K9me2 association in and around heterochromatic genes are consistent with those reported for HP1 by de Wit *et al.* [45,46]. These investigators used the DamID chromatin profiling technique and showed HP1 enrichment across the *lt*, *cta,* and *rl* heterochromatic genes in cultured cells. A direct association of HP1 and H3K9me with expressed heterochromatic gene sequences is consistent with models that evoke a facilitating role of heterochromatin-enriched proteins for heterochromatic gene expression [18]. Multifunctional roles for HP1, including in gene activation, are now widely acknowledged [47]. Our data show that H3K9me2 has, at minimum, a permissive role for heterochromatic genes. Other investigators reported recruitment of H3K9me3, which is generally associated with “silent heterochromatin” in mammals, as well as H3K9me2, to expressed euchromatic genes in mammalian cells [48,49]. The number of such examples is increasing with more comprehensive surveys of cell types [50,51]. Vakoc *et al.* [48,49] described complementary profiles of H3K9me3 and H3K9acet in active euchromatic genes which resembled those that we describe here, although H3K9me3 levels were far lower relative to levels of H3K9acet in these mammalian genes. H3K9me3 association with these genes is also highly dynamic and appears to correlate well with induced transcriptional activity and/or cell differentiation state, rather than repetitive DNA sequence content [48–51]. Our observations suggest pervasive H3K9me2 associated with active and inactive heterochromatic genes in embryos, except at the start regions, and the relatively high levels of H3K9me2 may be maintained by the high density of nearby repetitive DNAs that characterize heterochromatic but not euchromatic genes in flies. Notably, within the limited euchromatic regions surveyed by ChIP-chip, we also identified several euchromatic genes with enriched H3K9me2 profiles. These genes lacked prominent H3K9/14acet peaks at their 5′ ends and are poorly represented in available embryo EST and cDNA collections, indicating that they are not abundantly expressed in embryos. In one case, the H3K9me-enriched regions corresponded to the exon cassettes of *Dscam*, a gene which is known for its extraordinarily complex alternative splicing [52]. It is conceivable that H3K9me2 is involved in the regulation of *Dscam* alternative splicing, since there are other genes in which chromatin has been implicated in alternative splicing control [53]. Another possibility is that H3K9me2 association may simply be due to repetitiveness of similar exonic sequences within the gene. More extensive surveys are required to determine if H3K9me may be occurring more generally in *Drosophila* euchromatic genes, perhaps at levels below the sensitivity of our ChIP-chip assay, in order to understand the possible significance, if any, of H3K9me in euchromatin.

An important question to address is which *trans*-acting factor(s) is responsible for enrichment of H3K9me2 in heterochromatic genes and the eu-het transition zones. SU(VAR)3–9 was a reasonable candidate since it is the major HMTase responsible for H3K9me2 and HP1 recruitment in constitutive heterochromatin [24]. This role was based on the observation that the *Su(var)3–9^06^* null mutation results in strong reduction of H3K9me2 and HP1 immunostaining in the chromocenter, but not the euchromatin or Chromosome 4, in larval polytene nuclei [24,26]. Furthermore, *Su(var)3–9^06^* mutants show reduced levels of H3K9me2 relative to wild type levels in assays of bulk chromatin of 0–12 h embryos [24] or larvae [26], and reduced recovery of certain highly repetitive sequences in ChIP assays of larval [54] or adult [55] chromatin. Two features of the H3K9me2 profiles of *Su(var)3–9^06^* embryos reported here are remarkable in light of previous observations. First, the H3K9me2 landscapes in eu-het transition zones and in heterochromatic genes was overall similar in mutant and wild type embryos. Second, differences that were observed were relatively small local increases, rather than decreases of H3K9me2 in the mutant. These changes were evident as altered shapes of the H3K9me2 profiles across heterochromatic genes and Chromosome 4. This effect of *Su(var)3–9^06^* may be limited to embryos and/or the specific heterochromatic regions that we assayed. Our assays probed the single copy sequences interspersed within much larger expanses of repetitive DNA. The ChIP-chip probes were distributed across 1.35 Mb of Chromosome 4 and across 15 Mb of DNA located at the base of 2L, 2R, and 3L, including the eu-het transition zones and extending into the sequenced heterochromatin of these chromosome arms [17]. Thus, while we sampled across a significant proportion of the sequenced portions of Chromosome 4 and autosomal heterochromatin, the single copy probes themselves totaled 0.8 Mb, representing roughly 1%–2% of the estimated 59 Mb of heterochromatin present in diploid cells. Thus, the subtle effect of *Su(var)3–9^06^* detected in our ChIP assays would be easily missed in assays of bulk chromatin from embryos and masked by the pronounced effect of the mutation on H3K9me2 levels in tandemly repeated DNAs [24,56].

At minimum, our findings indicate that SU(VAR)3–9 is not responsible for the majority of H3K9me2 in heterochromatic genes and the eu-het transition zones in embryos. They also suggest that SU(VAR)3–9 influences one or more HMTases that act on heterochromatic genes and Chromosome 4 in embryos. The influence of SU(VAR)3–9 may be to limit the activities of other HMTases, perhaps through direct binding or by competing for shared factors or histone substrates. We do not know if the altered H3K9me2 profiles associated with *Su(var)3–9^06^* have functional consequences. We note that strong effects of *Su(var)3–9* mutations on heterochromatic gene expression have not yet been reported and are not expected since *Su(var)3–9^06^* flies are homozygous viable and fertile while many heterochromatic genes, including *lt* and *cta,* are essential for viability and/or fertility. Recent studies have identified DmSETDB1 as an HMTase which is primarily responsible for Chromosome 4 H3K9me2 in larvae and adults, although it appears to be acting independently of SU(VAR)3–9 at these stages [57]. dG9a has been characterized as a H3K9 HMTase with a partially overlapping function with SU(VAR)3–9 at multiple developmental stages [56]. Hence, these and perhaps other, as yet unidentified HMTase activities, may contribute more prominently than SU(VAR)3–9 to H3K9me2 dynamics in the heterochromatic regions that we assayed in embryos.

The characterization of the molecular landscape of modified histones along the long chromosome arms provided new insights into the contributions of TEs in influencing H3K9me distribution. We were surprised to see the abrupt increase of H3K9me2 enrichment in the euchromatin-heterochromatin transition zones. The striking coincidence of the transitions with marked increase in the density of retrotransposons in all three of the chromosome arms (2L, 2R and 3L) examined in this study suggests a mechanistic link. This is consistent with the finding that the TEs with highest average H3K9me2-enrichment in the ChIP-chip assay belong to the retrotransposon class (Figure S6). We also note an apparent overrepresentation of retrotransposon sequences in the list of cloned repeat-derived small RNAs [13] which have been implicated in heterochromatin formation [6]. Taken together, the results implicate clusters of retrotransposons as major determinants in the demarcation of H3K9me2-containing domains, and what may correspond to the distal edges of pericentric constitutive heterochromatin in *Drosophila* embryos.

An emerging concept from this and other studies is that it is not repetitive sequences *per se* that specifies heterochromatin but the physical proximity of multiple repetitive sequences of a certain type [45,58,59]. This contrasts with the idea of “cryptic heterochromatin,” suggested by Lippman *et al.* [44], in which isolated copies of TEs in euchromatin are viewed as heterochromatin because they bear the molecular markers generally associated with heterochromatin, including H3K9me2. In this study, numerous isolated euchromatic copies of retrotransposons did not bear marks of H3K9me2 and the TEs with high copy-numbers in euchromatin generally show low average H3K9me2-enrichment. If, for example, every copy of *roo* element in euchromatin was associated with H3K9me2, we would expect highly elevated ChIP-to-input signal ratios on the *roo* element microarray probes. Instead, the results of our studies are consistent with the notion that majority of the isolated copies of TEs in euchromatin are nucleating little, if any, H3K9me2 association, at least in embryonic chromatin.

The H3K9me2 landscape in the *T(2;3)lt^x13^* chromosome revealed complex redistribution of H3K9me2 featuring several peaks at discrete sites as far as a few megabases from the breakpoint. This provides molecular evidence for the impressive distances over which epigenetic effects of chromosome rearrangements can act in multicellular organisms. It is of interest to know why certain sites might attract the complexes that permit H3K9me2-enrichment. Inspection of the newly induced euchromatic sites in *T(2;3)lt^x13^* and euchromatic sites in normal chromosomes indicate that the two types of H3K9me2 peaks might be formed via similar mechanisms. In both cases, peaks are associated with gene exons or with clusters of duplicated genes, although sequence similarity among these duplicated segments are not necessarily extensive. Discovering additional examples should be informative to better understand the underlying basis for H3K9me enrichment. Comparisons of profiles in different chromosome sources should reveal if euchromatic sites are reproducible in different stages or tissues. Whatever determines the affinity for H3K9me2 at euchromatic sites, the analysis of the *T(2;3)lt^x13^* rearrangement has established that discrete sites in euchromatin can nucleate high-levels of H3K9me2 if situated in the appropriate physical context.

In summary, these studies of *Drosophila* heterochromatic genes and the euchromatin-heterochromatin transition zones challenge several traditional notions of the epigenetic signatures thought to distinguish heterochromatin from other chromatin domains. Instead, the results contribute to a growing body of evidence that suggests versatility in the activities of different types of heterochromatin-enriched repetitive DNA sequences and modified histones and emphasize the importance of chromosomal context. Knowledge of the modified histone distribution in and around heterochromatin and redistribution by chromosome rearrangement represents an important step toward understanding the function of epigenetic modifications in complex eukaryotic genomes.

## Materials and Methods

### Flies and antibodies.

D. melanogaster strains used in this study included *y; cn bw sp* (the strain used for whole genome sequencing), *T(2;3)lt^x13^*, and a wild-type *Canton-S* line which was the *T(2;3)lt^x13^* parental strain [19]. We also used the *w^m^* ^4^; *Su(var)3–9^06^* strain obtained from G. Reuter and verified that it carried the *Su(var)3–9^06^* insertion by PCR [26]. We used the following antibodies which have been previously used to detect histone modifications in *Drosophila*: Upstate (http://www.upstate.com/) antibodies #07–441 (anti-H3K9-di-methylation) [24,26], #07–030 (anti-H3K4-di-methylation) [30], #06–599 (anti-H3K9/K14-acetylation) [30], and Abcam (http://www.abcam.com/) #ab1791 (anti-H3 C-terminus) [60]. Quality controls verified that that the Upstate anti-H3K9me2 antibody acted as previously described in immunostaining assays of polytene chromosomes [24] and also in ChIP assays. For ChIP, we obtained enriched recovery of the 359bp-satellite repeat (4-fold and 8.9-fold in two separate trials) from immunoprecipitated relative to input embryonic chromatin using the PCR primers described by Rudolph *et al.* [55]

### Chromatin immunoprecipitation.

Detailed ChIP protocol is described in the Protocol S1. Briefly, 4–14 h embryos were subjected to cross-linking with 1% formaldehyde and chromatin was sheared with sonication. After immunoprecipitation with appropriate antibodies, DNA was purified from the chromatin and used for quantitative PCR or microarray hybridization. ChIP enrichment factors shown in Figure 1 were determined by comparing intensities of radiolabeled PCR fragments. Quantitative duplex PCRs were carried out as described by Noma *et al.* [12] and normalized using an arbitrarily chosen euchromatic control gene *Pdi*. The *Pdi* PCR fragment (bottom band) was 368 bp and the heterochromatin PCR fragments (top band) were 500–600 bp. Two or more independent immunoprecipitations were performed for each antibody and the error bars represent standard deviations. For the ChIP-chip assays shown in Figures 3–7 and S3–S6, immunoprecipitated DNA was amplified with two rounds of ligation-mediated PCR (23 and 6 cycles, respectively) essentially as described by Li *et al.* [61], or by GenomePlex Whole Genome Amplification Kit (Sigma, http://www.sigmaaldrich.com/), and used for microarray hybridization.

### Genome tiling array.

The tiling array contained sequences located in euchromatin-heterochromatin transition zones on chromosome arms 2L, 2R, and 3L and extending into the heterochromatin as assembled and annotated in R5.1 of the D. melanogaster genome [39]. It also included the sequenced portion of Chromosome 4 (1.35 Mb), a portion of distal 3R euchromatin (5.96 Mb, coordinates 21,932,592 to 27,897,863), and canonical sequences from a collection of 106 TEs. The TE sequences were from the archived set DMEL.TRANSPOSABLE.ELEMENTS.9.4.1. (downloaded from http://www.fruitfly.org/p_disrupt/datasets/ASHBURNER/D_mel_transposon_sequence_set.fasta [43, 62] and are described in Table S1).

The contigs of interest were extracted from TE-masked R5.1 *Drosophila* genomic sequence (downloaded from http://www.fruitfly.org/sequence/release5genomic.shtml). The probe set and microarray were custom-manufactured at NimbleGen (http://www.nimblegen.com/). Overlapping 50mer probes were designed with 40 bp resolution. In selecting the final set of probes for the array, only those probes with the 15mer-test score of 2.55556 or less were retained, meaning that 15mers contained in each 50mer probe appeared on average no more than 2.55556 times in the unmasked genome sequence. This is a highly stringent repeat-filtering compared to the customary cutoff score of up to 100. When an even more stringent cutoff (15mer-test score of 1.25 or less) was applied in the post-hybridization analyses, the overall profiles of H3K9me2 were not affected (Figure S6). The canonical TE collection was exempt from the repeat filtering and probes were designed to cover each TE. Sample labeling and hybridizations were carried out at NimbleGen using standard procedures. The ChIP/reference raw signal ratios were normalized by the medians of the entire array. Enrichment is expressed as fold enrichment relative to one of two reference controls, either total genomic DNA or anti-H3 C-terminus ChIP, which produced similar results (Figure S6).

## Supporting Information

Figure S1 Transcription of 2Lh Genes in Embryos as Determined by Nuclear Run-On Assay Plasmids containing *lt* (1), *cta* (2), *Chitinase* (3), and *snky* (4) cDNAs were digested with restriction enzymes to separate cDNA and vector sequences. The top panel shows restriction fragments separated on an agarose gel and stained with ethidium bromide (top panel). Asterisks in each lane indicate bands containing vector sequences. A Southern blot prepared from this gel was hybridized with newly synthesized radioactive RNAs from nuclei isolated from 4–14 h embryos. Hybridization signals (bottom panel) verify expression of *lt* and *cta* and weak expression of *Chitinase*. *snky* is expressed only in the testis [63] and served as a negative control.
(596 KB EPS)

Figure S2 Verification of Specificity of the *light* Gene PCR Primer Pairs The figure shows gel assays demonstrating that *lt* primer sets (sets 1–10 on Figure 1A) amplified fragments of expected length from genomic DNA of normal embryos (left panel) but not from DNA from embryos homozygous for a *lt* deletion (right panel). To obtain deletion homozygotes, which die before hatching, *Df(2L)lt^x64^*/*CyO*-*GFP* adults were mated, their embryos were collected for 10–16 h and classified as control *CyO* embryos (identified as GFP-positive) or *Df(2L)lt^x64^* homozygotes (identified by lack of GFP expression). Genomic DNA extracted from individual embryos was used for duplex-PCR with a primer set for the *lt* region and a primer set for *Pdi,* a euchromatic gene located outside of *Df(2L)lt^x64^.* PCR products were assayed after gel electrophoresis and ethidium bromide staining. Left panel shows that in GFP positive embryos, PCR products were detected for *lt* primers (top band, with lane numbers 1–10 corresponding to primer sets shown in Figure 1A) and control *Pdi* primers (bottom band). Right panel shows that in *Df(2L)lt^x64^* homozygotes, *lt* primers do not produce a PCR product but *Pdi* primers do. For both panels, the *ck* lane is a positive control in which primers for another euchromatic control gene (*crinkled*, top band) and *Pdi* primers (bottom band) were used; H_2_O is a negative control (with *Pdi* and *ck* primers*,* no DNA template added). The *Df(2L)lt^x64^* and *CyO*-GFP chromosomes were described previously [19,64].
(792 KB EPS)

Figure S3 H3K9me2 Landscape in Chromosome 4 The figure compares the H3K9me2 profile of 3R, which is even and typical of euchromatin in (A) to the Chromosome 4 profile, which has pervasive H3K9me2 association slightly above the baseline level in (B). The two regions are of equal size (∼1.3 Mb) and drawn to scale. Profiles are from *y; cn bw sp* and similar profiles were observed and other wild type genotypes. The particularly outstanding peak on Chromosome 4 corresponded to the coding region of the *bent* gene (coordinate 0.7–0.8 Mb). Annotation of the Chromosome 4 uses symbols as described for Figure 6D–6F, except that the window size of the moving average is 50 probes. The baseline (enrichment factor = 1.0) is shown in gray. Due to this H3K9me2 association throughout the chromosome and the equally even distribution of the *1360* elements, it was difficult to make a correlation between H3K9me2 peaks and the *1360* elements or any other TEs. Sites of insertion of variegating (speckled arrowheads) and non-variegating transgenes (black arrowheads) as reported by Sun *et al.* [16] and based on adult eye color are shown above the H3K9me2 profile. A cursory inspection did not indicate a striking correlation of variegated transgenes with elevated H3K9me2 levels, although it should be noted that adult eye pigmentation was used to define silenced transgenes [16], while these ChIP experiments assayed embryonic chromatin. Locations of *1360* elements are indicated by the taller bars among the DNA TEs.
(C) Comparison of H3K9me2 profile across Chromosome 4 of wild type (black) and *Su(var)3–9^06^* (red) embryos. The arrow marks the peak associated with the *bent* gene. This peak is similar in the two genotypes but is surrounded by localized increases in H3K9me2 levels in the mutant compared to wild type embryos.
(1.0 MB EPS)

Figure S4 Close Up Views of the Euchromatic Genes Associated with the Outstanding H3K9me2 Peaks Shown in Figure 6A–6C In each panel, shown at the top are non-smoothed H3K9me2-enrichment values relative to the H3-ChIP reference control, and at the bottom are the genes with the depiction of exon/intron structures. Where present, TEs are shown in gray. For *Dscam* gene, the alternatively spliced exon cassettes are bracketed. *Dscam* alternative exon 4s are 159–171 bp each in length and up to 81% identical to each other at the nucleotide sequence level.
(972 KB EPS)

Figure S5 H3K9me2 Association with TEs (A) Examples of H3K9me2 profiles in individual TEs in two different genetic backgrounds. Genotypes are noted at the top and TE names below the profiles. The H3K9me2-enrichment is noted along length of the TE (*x*-axis) and enrichment factor on the *y*-axis.
(B) The 106 TEs present on the microarray were ranked according to the average H3K9me2-enrichment values along their lengths and aligned from highest (left) to lowest (right) on the graph. The color-coding is the same in Figure 1: dark blue, LTR retrotransposon; light blue, LINE-like retrotransposon; red, DNA transposon; pink, INE-1 (arrow). The six TEs shown in (A) are marked by asterisks. Where available, the copy number for each TE in euchromatin [43] is indicated by rectangles using the copy number scale on the right *y*-axis. For a complete list of TEs and enrichment values, see Table S1.
(1.7 MB EPS)

Figure S6 Reproducibility of the H3K9me2 Profiles Obtained by ChIP-chip Comparison of Chromosome 2R profiles in which (A) H3K9me2-enrichment expressed relative to the genomic DNA reference control in the *T(2;3)lt^x13^* strain, and (B) H3K9me2-enrichment expressed relative to the anti-H3 C-terminus ChIP reference control in *y; cn bw sp* strain. There are examples of strain-differences (most noticeable here is the *T(2;3)lt^x13^* strain-specific peak at the coordinate 1.7 Mb), and peak heights could also vary considerably between the experiments. When anti-H3 C-terminus ChIP sample was used as a reference control, intended to account for nucleosome density differences [65], numerous low- to medium-height peaks became apparent (three examples are marked by asterisks in [B]). Close examinations revealed that virtually all of them were located at the 5′-ends of expressed genes (e.g., see *CG10600*, *CG5274*, and *Dscam* genes in Figure S4). We note that the baseline for H3K9me2-ChIP is set by the regions free of this modified histone (i.e., non-specifically precipitated DNA), whereas the baseline for H3-ChIP is set by the regions evenly occupied by nucleosomes. Thus, we believe these conditional peaks simply reflect the depletion of nucleosomes at the promoter [66–68] and not necessarily occurrence of H3K9me2. That is, when expressed as the H3K9me2-ChIP/H3-ChIP ratios, the numerator remained at the baseline whereas the denominator became reduced. Aside from these peripheral variations, however, the overall H3K9me2 profiles were highly reproducible regardless of the strains and the types of reference control. In particular, the positions of the “edge” of the H3K9me2-enriched domains were invariable in all experiments.
(C) Is the same experiment as (B), but even more stringent cutoff (i.e., 15mer-test score of 1.25 or below) was applied in the post-hybridization analysis to further eliminate probes with a potential of cross-hybridization. The profiles remained essentially the same.
(2.3 MB EPS)

Protocol S1 Detailed Protocols for Nuclear Run-on and ChIP Assays (21 KB RTF)

Table S1 Transposable Elements Assayed by ChIP-chip (181 KB RTF)

### Accession Numbers

The data discussed in this manuscript have been deposited in the National Center for Biotechnology Information's (NCBI) Gene Expression Omnibus (GEO, http://www.ncbi.nlm.nih.gov/geo/) and are accessible through GEO Series accession number GSE7839.

## Abbreviations:

ChIP: chromatin immunoprecipitation
H3K9me2: histone H3 di-methylated at lysine 9
HMTase: histone methyltransferase
TE: transposable element
