# Supplementary material for: Molecular Landscape of Modified Histones in Drosophila Heterochromatic Genes and Euchromatin-Heterochromatin Transition Zones
Source: PLoS Genet. 2008 Jan 18;4(1):e16. doi: 10.1371/journal.pgen.0040016 (PMC2211541; doi:10.1371/journal.pgen.0040016)
Supplement: Supplementary file 1 [file pgen.0040016.sd001.rtf]

Supporting Protocols
Nuclear run-on assay.  The nuclear run-on assay protocol was adapted from Hirayoshi and Lis [69]. Four to fourteen hour old embryos (y; cn bw sp strain) were collected, dechorionated, and washed with 0.03% Triton X-100, 0.4% NaCl.  Embryos were homogenized in 7.5 ml buffer A (300 mM sucrose, 2 mM magnesium acetate, 3 mM CaCl_2_, 10 mM Tris-HCl (pH 8.0), 0.1% Triton X-100, 0.5 mM DTT) using a tight-fit glass pestle for 10 strokes on ice. The homogenate was centrifuged first at 550 g for 1 min to remove debris and then at 5,000 g for 10 min to pellet crude nuclei. Pellets were suspended in 10 ml buffer A/B mix (50%/50%) (buffer B: 2M sucrose, 5 mM magnesium acetate, 10 mM Tris-HCl (pH 8.0), 0.5 mM DTT) and layered on the same volume of buffer B. After centrifugation at 18,000 g for 20 min in a swing-bucket rotor, supernatant was removed by aspiration and the nuclei pellets were suspended in 1 ml buffer D (25% glycerol, 5 mM magnesium acetate, 50 mM Tris-HCl (pH 8.0), 0.1 mM EDTA, 5 mM DTT), spun down in a microcentrifuge for 20 sec, and resuspended in 100 l buffer D. This stock was frozen with liquid nitrogen and stored at –80C. To carry out the run-on reaction, the nuclear suspension was mixed with 114 l reaction buffer (7.2 mM Tris-HCl (pH 8.0), 4.4 mM magnesium acetate, 1.4 mM MnCl_2_, 150 mM KCl, 3 mM each of ATP, CTP and GTP, 10 M UTP, 100 Ci -^32^P UTP (3000 Ci/mmol) and 1 unit RNasin) and incubated at room temperature for 30 min. The reaction was stopped by addition of 1 l DNase I (Invitrogen) and incubation at 37C for 10 min. RNA was isolated by using the Ultraspec reagent, dissolved in 200 l TE, and denatured at 100C just before hybridization. 
	To prepare the Southern blot, 3 g each of the cDNA plasmid clones was digested with appropriate restriction enzymes to separate the cDNA insert and the vector backbone, electrophoresed in an agarose gel, stained with ethidium bromide and transferred to a nylon membrane using the standard methods [70]. The light and sneaky cDNA clones were described previously [31,63]. Full-length concertina and Chitinase cDNA clones were obtained from the Drosophila Genomics Resource Center. The blot was trimmed to 20 cm^2^ and pre-hybridized overnight in 10 ml hybridization solution (6X SSC, 50% (v/v) formamide, 1% (w/v) SDS, 10% (w/v) dextran sulfate, 250 g/ml salmon sperm DNA) at 42C. Hybridization was carried out in 2 ml hybridization solution with 510^6^ cpm RNA probe at 42C for 48 hours. The blot was washed extensively with 2X SSC, 0.1% SDS at room temperature and then 0.2X SSC, 0.1% SDS at 60C. The radioactive signal was visualized using a phosphorimager. 

Chromatin immunoprecipitation.  The ChIP protocol was adapted from Cavalli et al. [71] and Ekwall and Partridge [72]. Drosophila embryos were collected using fly cages furnished with grape-juice agar plates. Approximately 500 l of 4-14 hour old embryos were dechorionated with 50% bleach for 90 sec and washed extensively with 0.03% Triton X-100, 0.4% NaCl solution. The embryos were washed again with 50 ml 0.01% Triton X-100 in PBS and suspended in 10 ml cross-linking solution (50 mM HEPES pH 7.6, 1 mM EDTA, 0.5 mM EGTA, 100 mM NaCl, 1% formaldehyde). The embryo suspension in a 50 ml conical tube was topped off with 30 ml n-heptane and shaken vigorously for 15 min. The embryos were then spun down in a table-top centrifuge for 30 sec and resuspended in 50 ml stop solution (0.125M glycine, 0.01% Triton X-100 in PBS). The embryos were washed twice with 10 ml wash solution (10 mM HEPES pH 7.6, 10 mM EDTA, 0.5 mM EGTA, 0.25% Triton X-100) for 10 min each with gentle shaking and finally suspended in 3 ml lysis buffer (50 mM HEPES pH 7.6, 140 mM NaCl, 1 mM EDTA, 1% Triton X-100, 0.1% sodium deoxycholate) supplemented with protease inhibitors (1 mM PMSF and 'complete' protease inhibitor cocktail (Roche)). The embryo suspension was sonicated in the presence of 0.5 g glass beads (0.1 mm diameter) using Misonix model 3000 equipped with microtip 418 (setting 10 for 30 sec  10 times). Under these conditions, the majority of the genomic DNA fragments were < 1 kb as assayed on agarose genes, although a light smear extending up to 5 kb persisted even after extensive sonication. The fragmented chromatin was centrifuged twice (15,000 g, 5 min and 15 min) in a microcentrifuge, passed through 0.8 m filter to remove insoluble material, then stored at –80C.
	Five hundred l sheared chromatin was incubated with 60 l of a slurry of Protein A-Sepharose (Sigma) (50% v/v in TBS) for 1 hour with gentle shaking in a cold room to block non-specific binding and centrifuged at 15,000 g for 5 min to remove the Sepharose beads. At this point, 50 l was set aside for 'input' sample. Five to ten g antibody was then added to the rest of chromatin and it was incubated overnight in a cold room with gentle shaking. The next day 60 l Protein A-Sepharose slurry was added and incubation was continued for another 2 hours. The Sepharose beads were collected by centrifugation for 2 min and washed for 5 min each with 1 ml each of the following solutions: lysis buffer (twice), lysis buffer with 500 mM NaCl, LiCl buffer (250 mM LiCl, 10 mM Tris-HCl (pH 8.0), 1 mM EDTA, 0.5% Igepal CA-630, 0.5% sodium deoxycholate), and TE, each time followed by 2 min centrifugation and removal of the solution. The beads were finally suspended in 50 l TES (50 mM Tris-HCl (pH 8.0), 10 mM EDTA, 1% SDS) and incubated at 65C for 10 min to release the chromatin. After recovering the supernatant, the beads were resuspended in 200 l TES, spun for 1 min, and the supernatants were pooled. The 'input' sample was also supplemented with 200 l TES and treated in parallel from here on. The chromatin was incubated overnight at 65C to reverse the cross-linking. Two-hundred and fifty l TE and 25 l proteinase K (10 mg/ml) were then added and the sample was incubated at 37C for 2 hours. After phenol/chloroform extraction, DNA was ethanol-precipitated, dissolved in 100 l TE with 10 g RNase A, and incubated at 37C for 30 min. 
	For the quantitative PCR assay, the 'input' sample was diluted in TE (typically 200 fold) and the ChIP sample was usually not diluted. Duplex PCR was done in 40 l volume containing 3 l DNA solution and 0.2 l -^32^P dCTP (10 Ci/l, 3000 Ci/mmol). Pilot experiments indicated that the cycle number had to be as low as 26 to obtain linearly proportional quantification. However, we used the cycle number of 30 because it yielded the most reliable relative quantification for all primer pairs used in this study. Five l of the completed PCR reaction was electrophoresed in 5% acrylamide gel in TBE. The gel was dried and the radioactivity of the bands was quantified using a phosphorimager. 

References 
69. Hirayoshi K, Lis JT (1999) Nuclear run-on assays: assessing transcription by measuring density of engaged RNA polymerases. Methods Enzymol 304: 351-362.
70. Sambrook J, Russell DW (2001) Molecular Cloning:  A laboratory manual: Cold Spring Harbor Laboratory Press.
71. Cavalli G, Orlando V, Paro R (1999) Mapping DNA target sites of chromatin-associated proteins by formaldehyde cross-linking in Drosophila embryos. In: Bickmore W, editor. Chromosome Structural Analysis: A Practical Approach. Oxford: Oxford University Press. pp. 21-37.
72. Ekwall K, Partridge JF (1999) Fission yeast chromosome analysis: fluorescence in situ hybridization (FISH) and chromatin immunoprecipitation (CHIP). In: Bickmore W, editor. Chromosome Structural Analysis: A Practical Approach. Oxford: Oxford University Press. pp. 39-57.
