# Supplementary material for: Molecular Landscape of Modified Histones in Drosophila Heterochromatic Genes and Euchromatin-Heterochromatin Transition Zones
Source: PLoS Genet. 2008 Jan 18;4(1):e16. doi: 10.1371/journal.pgen.0040016 (PMC2211541; doi:10.1371/journal.pgen.0040016)
Supplement: Supplementary file 2 [file pgen.0040016.st001.rtf]

Table S1.  Transposable Elements Assayed by ChIP-chip

Accession Number | Element^1^
	Average  H3K9me2 Enrichment^2^	Element type^1^
	Copy # in Euchromatin^1^	
gb|X06950|G_element	2.788598926	LINE-like retrotransposon	3	
gb|AY089271|Osvaldo	2.628224232	LTR retrotransposon	N/A	
gb|AE003788|gypsy8	2.545014729	LTR retrotransposon	N/A	
gb|AB022762|aurora_element	2.466987906	LTR retrotransposon	3	
gb|nnnnnnnn|diver2	2.460091875	LTR retrotransposon	9	
gb|nnnnnnnn|Rt1c	2.419143621	LINE-like retrotransposon	1	
gb|nnnnnnnn|gypsy6	2.275002205	LTR retrotransposon	1	
gb|AL035631|gypsy2	2.240099146	LTR retrotransposon	3	
gb|nnnnnnnn|invader1	2.187770606	LTR retrotransposon	26	
gb|nnnnnnnn|Doc3_element	2.170000794	LINE-like retrotransposon	9	
gb|U14101|TART_B	2.126318804	LINE-like retrotransposon	N/A	
gb|nnnnnnnn|gypsy10	2.123822366	LTR retrotransposon	N/A	
gb|AE002591|gypsy9	2.120596102	LTR retrotransposon	N/A	
gb|NT_033778|invader6	2.112770706	LTR retrotransposon	N/A	
gb|AJ010298|GATE	2.093136453	LTR retrotransposon	20	
gb|AY600955|TART_C	2.092548714	LINE-like retrotransposon	N/A	
gb|X51968|R1A1_element	2.087741295	LINE-like retrotransposon	0	
gb|AJ000387|ZAM	2.087182860	LTR retrotransposon	0	
gb|X51967|R2_element	2.077842373	LINE-like retrotransposon	0	
gb|nnnnnnnn|Helena	2.072803677	LINE-like retrotransposon	7	
gb|nnnnnnnn|baggins	2.071490739	LINE-like retrotransposon	14	
gb|nnnnnnnn|Doc2_element	2.070831966	LINE-like retrotransposon	1	
gb|AC006215|G2	2.065422975	LINE-like retrotransposon	14	
gb|nnnnnnnn|G3	2.061004989	LINE-like retrotransposon	4	
gb|U23420|3S18	2.047786445	LTR retrotransposon	6	
gb|AJ487856|Max_element	2.043457843	LTR retrotransposon	N/A	
gb|X07656|1731	2.042857967	LTR retrotransposon	2	
gb|nnnnnnnn|Circe	2.029597796	LTR retrotransposon	2	
gb|nnnnnnnn|gypsy4	2.025017239	LTR retrotransposon	2	
gb|AF237761|X_element	2.003342077	LINE-like retrotransposon	25	
gb|nnnnnnnn|accord	1.992414729	LTR retrotransposon	1	
gb|AC007477|gypsy3	1.987017881	LTR retrotransposon	2	
gb|AE003788|gypsy7	1.974706167	LTR retrotransposon	N/A	
gb|X15066|micropia	1.948235445	LTR retrotransposon	5	
gb|AE003789|gypsy12	1.944476626	LTR retrotransposon	N/A	
gb|nnnnnnnn|Dm88	1.938702076	LTR retrotransposon	32	
gb|AC005734|Rt1b	1.931080401	LINE-like retrotransposon	37	
gb|AJ278684|Rt1a	1.926537210	LINE-like retrotransposon	13	
gb|M12927|gypsy	1.917171626	LTR retrotransposon	2	
gb|nnnnnnnn|invader4	1.877291287	LTR retrotransposon	9	
gb|nnnnnnnn|Stalker3T	1.870330381	LTR retrotransposon	N/A	
gb|nnnnnnnn|Ivk	1.854219718	LINE-like retrotransposon	7	
gb|nnnnnnnn|invader3	1.831292594	LTR retrotransposon	16	
gb|AF492763|frogger	1.814522076	LTR retrotransposon	1	
gb|nnnnnnnn|rooA	1.800129258	LTR retrotransposon	5	
gb|AF365402|HMS_Beagle2	1.777215922	LTR retrotransposon	N/A	
gb|AC005198|F_element	1.777078612	LINE-like retrotransposon	42	
gb|nnnnnnnn|Cr1a	1.761564842	LINE-like retrotransposon	56	
gb|nnnnnnnn|transib3	1.757080280	DNA transposon	7	
gb|AE002612|Q_element	1.754797644	LTR retrotransposon	N/A	
gb|AC006215|gtwin	1.753821778	LTR retrotransposon	6	
gb|AY561850|TART_A	1.734373159	LINE-like retrotransposon	1	
gb|AF541947|accord2	1.727265571	LTR retrotransposon	1	
gb|nnnnnnnn|BS	1.634203005	LINE-like retrotransposon	29	
gb|nnnnnnnn|invader2	1.609462065	LTR retrotransposon	10	
gb|nnnnnnnn|transib2	1.606450303	DNA transposon	12	
gb|AJ542581|TAHRE	1.605805118	LINE-like retrotransposon	N/A	
gb|AF541948|McClintock	1.603561625	LTR retrotransposon	2	
gb|AF364549|springer	1.595772197	LTR retrotransposon	11	
gb|X17551|Doc	1.583664649	LINE-like retrotransposon	55	
gb|AF420242|Stalker	1.579328533	LTR retrotransposon	12	
gb|AF492764|rover	1.578296418	LTR retrotransposon	6	
gb|nnnnnnnn|gypsy11	1.552884736	LTR retrotransposon	N/A	
gb|AF541949|Stalker4	1.551451217	LTR retrotransposon	2	
gb|nnnnnnnn|Tc1	1.548605056	DNA transposon	21	
gb|U06920|HeT_A	1.540979263	LINE-like retrotransposon	3	
gb|X95908|mdg3	1.533327763	LTR retrotransposon	16	
gb|AC004377|diver	1.525789382	LTR retrotransposon	9	
gb|nnnnnnnn|mariner2	1.492096551	DNA transposon	17	
gb|AJ009736|Idefix	1.486743016	LTR retrotransposon	7	
gb|AC007146|Tabor	1.485850934	LTR retrotransposon	2	
gb|nnnnnnnn|invader5	1.484067607	LTR retrotransposon	6	
gb|AY180918|opus	1.481467488	LTR retrotransposon	24	
gb|nnnnnnnn|transib4	1.476864304	DNA transposon	5	
gb|nnnnnnnn|S2	1.476153041	DNA transposon	13	
gb|AC005453|1360	1.475729395	DNA transposon	105	
gb|nnnnnnnn|transib1	1.474870806	DNA transposon	2	
gb|Z27119|flea	1.465852607	LTR retrotransposon	17	
gb|U33463|S_element	1.433872493	DNA transposon	51	
gb|nnnnnnnn|Stalker2	1.426182357	LTR retrotransposon	13	
gb|AF541950|hopper2	1.418119767	DNA transposon	5	
gb|AF365402|HMS_Beagle	1.415876544	LTR retrotransposon	13	
gb|U66884|INE_1	1.392967358	INE1	N/A	
gb|X01472|17.6	1.373808150	LTR retrotransposon	12	
gb|X59545|mdg1	1.358180962	LTR retrotransposon	25	
gb|U89994|Burdock	1.349973352	LTR retrotransposon	13	
gb|AE003485|gypsy5	1.343830190	LTR retrotransposon	2	
gb|X80025|hopper	1.319604966	DNA transposon	15	
gb|AF364550|Quasimodo	1.307864988	LTR retrotransposon	14	
gb|AF222049|Transpac	1.298760276	LTR retrotransposon	5	
gb|AY180916|blood	1.255371031	LTR retrotransposon	22	
gb|AC009537|Tc3	1.241134193	DNA transposon	N/A	
gb|X59837|pogo	1.235296039	DNA transposon	44	
gb|X01748|HB	1.211040267	DNA transposon	32	
gb|M14954|I_element	1.208640370	LINE-like retrotransposon	28	
gb|V00246|FB	1.200276519	DNA transposon	32	
gb|AY180919|Juan	1.198339572	LINE-like retrotransposon	9	
gb|X02599|copia	1.197806166	LTR retrotransposon	30	
gb|X03431|297	1.166193012	LTR retrotransposon	57	
gb|nnnnnnnn|Tirant	1.133888774	LTR retrotransposon	20	
gb|nnnnnnnn|412	1.130867472	LTR retrotransposon	31	
gb|X67681|Bari1	1.108989317	DNA transposon	5	
gb|M69216|hobo	1.090091248	DNA transposon	24	
gb|AY180917|roo	1.070857264	LTR retrotransposon	146	
gb|X15469|NOF	1.058316350	DNA transposon	7	
gb|M22874|jockey	0.963597887	LINE-like retrotransposon	69	

^1^ Information from Kaminker et al. [43]
^2^ ChIP-chip data from the y; cn bw sp strain
